# Identification of Six Novel Variants of *ACAD8* in Isobutyryl-CoA Dehydrogenase Deficiency With Increased C4 Carnitine Using Tandem Mass Spectrometry and NGS Sequencing

**DOI:** 10.3389/fgene.2021.791869

**Published:** 2022-01-28

**Authors:** Dan-Yan Zhuang, Shu-Xia Ding, Fei Wang, Xiang-Chun Yang, Xiao-Li Pan, You-Wei Bao, Li-ming Zhou, Hai-bo Li

**Affiliations:** ^1^ The Central Laboratory of Birth Defects Prevention and Control, Ningbo Women and Children’s Hospital, Ningbo, China; ^2^ Department of Endocrinology and Genetic Metabolism of Pediatrics, Ningbo Women and Children’s Hospital, Ningbo, China; ^3^ Reproductive Medicine Centre, Ningbo Women and Children’s Hospital, Ningbo, China

**Keywords:** IBDHD, tandem mass spectrometry, ACAD8, genetic mutations, inherited metabolic diseases

## Abstract

Isobutyryl-CoA dehydrogenase deficiency (IBDHD, MIM: #611283) is a rare autosomal recessive hereditary disease, which is caused by genetic mutations of acyl-CoA dehydrogenase (ACAD) 8 and associated with valine catabolism. Here, tandem mass spectrometry (MS/MS) was applied to screen 302,993 neonates for inherited metabolic diseases (IMD) in Ningbo of China from 2017 to 2020. The results suggest that 198 newborns (0.7‰) were initially screened positive for IBDHD with C4-Carnitine, and 27 cases (0.1‰) were re-screened positive. Genetic diagnosis was performed on 21 of the 27 cases. Seven compound heterozygous variations, three biallelic variations, and one heterozygous variation of ACAD8 were found with a pathogenicity rate of 33.3% (7/21). In addition, seven biallelic variations, one heterozygous variation of acyl-CoA dehydrogenase short chain (*ACADS*), and one biallelic variation of acyl-CoA dehydrogenase short/branched chain (*ACADSB*) was detected. Further research showed that *ACAD8* mutations of 11 IBDHD cases distributed in six different exons with total 14 mutation sites. Five of which were known suspected pathogenic sites (c.286G > A, c.553C > T, c.1000C > T, c.409G > A, c.500del) and six were novel mutation sites: c.911A > T, c.904C > T, c.826G > A, c.995T > C, c.1166G > A, c.1165C > T. This finding enriched the mutation spectrum of *ACAD8* in IBDHD.

## 1 Introduction

IBD (isobutyryl-CoA dehydrogenase) is a mitochondrial enzyme that catalyzes the conversion from isobutyryl-CoA to methacryloyl-CoA in valine catabolism (Roe et al., 1998a; Battaile et al., 2004; Lin et al., 2018). IBD is encoded by *ACAD8* (MIM 604773) located on chromosome 11q25 (Nguyen et al., 2002). The *ACAD8* gene is composed of 11 exons, encoding 415 amino acids, consisting of the NH2 terminal a-helix domain, the inner b-chain domain and the C-terminal a-helix domain (Nguyen et al., 2002; Yoo et al., 2007). The amino acid sequence and overall structure of the ACAD8 protein are similar to other members of the ACD family (Telford et al., 1999). So far, most of the published *ACAD8* gene variants occur in exons 4 and 9 (Lin et al., 2018). Homozygous or compound heterozygous mutations in the *ACAD8* gene often cause IBDD, which is a rare autosomal recessive genetic metabolic disease. Since it was first reported in 1998 (Roe et al., 1998b), the prevalence and clinical significance of the disease have remained unclear, and the natural course of the disease is still unclear, and most patients are asymptomatic.

Symptoms of IBDD generally appear in late infancy or childhood, and symptoms include poor feeding, developmental delay, dilated cardiomyopathy, epilepsy, and anemia (Lin et al., 2018). The latest study found that a case of IBDD with obvious clinical symptoms in adulthood suggests that asymptomatic children with IBDD are at risk of clinical manifestations in adulthood (Nygaard et al., 2016a). In addition, another study found that alternative splicing of *ACAD8* caused mitochondrial defects and progressive liver steatosis in mice, suggesting a correlation between IBDHD and fatty liver (Sabbagha et al., 2011). Therefore, the clinical importance of IBDHD is unclear. The systematic evaluation of this disease is particularly urgent, and patients with IBDHD should be carefully monitored.

Tandem mass spectrometry (MS/MS) is a technology developed in recent years for screening inherited metabolic disorders (Chace et al., 1993). It can perform a single test on dry filter paper blood specimens within 2–3 min now. More than 30 genetic metabolic diseases with dozens of small molecule metabolites including amino acid, organic acid and oxidative fatty acid can be detected at one time (Pedersen et al., 2006; Santra et al., 2017; Lin et al., 2018). Now, A variety of inherited metabolic diseases of neonatus can be screened using tandem mass spectrometry in a single injection (Roe et al., 1998a; Battaile et al., 2004; Lin et al., 2018). Due to cross-over metabolic pathways, acylcarnitine analysis allowed the identification of many metabolic diseases. By detection of C4-butyrylcarnitine or isobutyrylcarnitine, short-chain acyl-CoA dehydrogenase deficiency (SCADD [MIM 606885]) (Koeberl et al., 2003), isobutyryl-CoA dehydrogenase deficiency (IBDHD [(MIM 611283]) (Lin et al., 2018), and ethylmalonic encephalopathy (EE [MIM 201470]) (Forni et al., 2010) can be assessed. In addition, acylcarnitine is also a valuable marker for diagnosis of glutaric acidemia type II (GA2 [MIM 213680]) (Forni et al., 2010; Sadat et al., 2020).

This study summarized the results of neonatal genetic and metabolic disease screening in Ningbo from 2017 to 2020. Tandem mass spectrometry technology combining with urine gas chromatography mass spectrometry and sequencing technology were used to analyze the clinical and genetic characteristics of IBDD. Through NBS program and DNA analysis, we demonstrated the pathogenicity of *ACAD8* gene mutations and identified seven novel possible pathogenic sites.

## 2 Materials and Methods

### 2.1 Patient Cohort

In 2017–2020, 302,993 newborns were screened for genetic and metabolic diseases at the Center for Birth Defects Prevention and Control laboratory in Ningbo. Informed consents were signed prior to the screening. Inclusion criteria were: i. Neonates born within 28 days in Ningbo; ii. The parents of the Neonates voluntarily consented to tandem mass spectrometry screening; iii. Follow up to the end of the project. Exclusion criteria were: i. Those who do not meet the entry criteria; ii. No dry blood points larger than 8 mm in diameter can be provided; iii. Because the assisted reproduction (third-generation test tube) through preimplantation genetic screening, they are already selective embryos, and the statistical rate or incidence of regional diagnosis is not accurate, so the pregnancy (including IVF-ET, ICSI pregnancy) patients who assisted preimplantation genetic screening (PGS) is excluded.

### 2.2 Sample Requirements

Prospective samples require two or more dried blood spots with a diameter of 8 mm for all incoming samples. At least 2 ml ofperipheral blood (EDTA anti-coagulation) was collected from the recovered sample. Withdrawal situation during the implementation of the project were: i. The subject guardian (either of the parents) voluntarily requested withdraw; ii. Parents did not cooperate with blood sampling when the screening indexes of the subject tandem mass spectrometry were abnormal.

### 2.3 Newborn Screening

Blood drops from the heel of 3–5 days old neonates were collected and dried on SS903 filter paper (Whatman) for examination. The amino acids and acylcarnitine in dried blood filter paper were determined by TQD tandem mass spectrometry (Waters) and the non-derivatized multiple amino acid, carnitine and succinylacetone determination kit (PerkinElmer).

Newborns with C4 screening index >0.51 μmol/L were recalled for MS/MS testing and urine gas chromatography mass spectrometry (GC-MS).

### 2.4 Genetic Testing

With informed consent, peripheral blood was collected from parents and newborns using the OMEGA Genomic DNA Extraction Kit (OMEGA Biotech, United States). The genomic DNA of the sample is extracted as the test material. Targeted sequencing was performed by the basic edition panel of inherited metabolic diseases (Genuine Diagnostic Laboratory, Hangzhou, China) to detect 94 genes, including ACAD8, PAH, ACADS, ACADSB MUT and other genes. The sequences of target regions were enriched by multiple probe hybridizations using Agilent SureSelect Human Exon Sequence Capture Kit. The capture products were then purified using Agencourt AMPure XP beads (Beckman Coulter). After purification and quality test, the sequencing libraries were quantified by Illumina DNA standard and Primer Premix Kit (kapa), and then massively parallel sequenced by Illumina MiSeq platform. All potentially pathogenic variants were verified by Sanger sequencing using the specific primers. PCR (polymerase chain reaction) conditions were according to TaKaRa LA PCR™ Kit Ver.2.1 (TaKaRa).

Suspected pathogenic mutations were obtained by consulting OMIM (https://omim.org/), ClinVar (https://www.ncbi.nlm.nih.gov/clinvar/), the Human Gene Mutation Database (http://www.hgmd.cf.ac.uk/ac/index.php), 1,000 Genome Project database (http://www.1000genomes.org/), ExAC consortium (http://exac.broadinstitute.org/), gnomAD (https://gnomad.broadinstitute.org/), laboratory internal database and literature. The novel missense variants were further assessed for possible pathogenicity based on tools including SIFT, PolyPhen-2, and MutationTaster.

### 2.5 Follow-Up

Follow-up was carried out through the Ningbo Maternal and Child Electronic Monitoring System. The monitoring items included physical examination, assessment of physical and mental development, followed up for 6–42 months. The assessment is carried out by a pediatrician. The assessment includes the physical growth and development of the child (including weight, height, head circumference, etc.), the development of the skull and teeth, as well as the development of the nervous system, movement and language. After the diagnosis of IBDD or SCADD, symptomatic treatment is mainly for the symptoms secondary to carnitine deficiency, and the clinical significance of most diagnosed cases is not clear; children diagnosed through the neonatal screening program, mainly through clinical follow-up monitoring of carnitine levels.

## 3 Results

### 3.1 Screening Results

**FIGURE 1:**
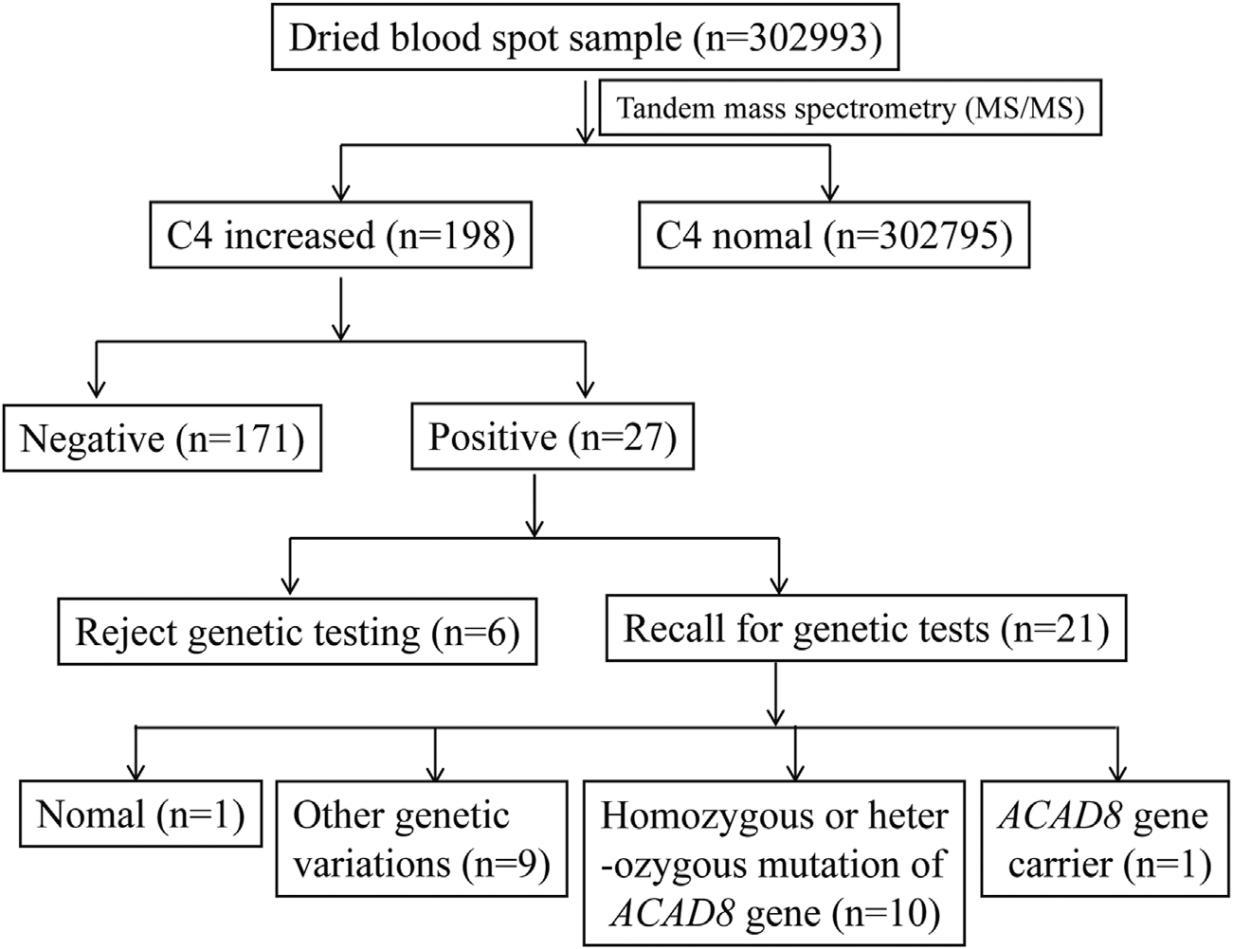
Flow chart of newborn screening and diagnosis.

A total of 302,993 newborns in Ningbo area were screened for genetic metabolic diseases from 2017 to 2020. Among them, 198 cases showed elevated levels of C4 acylcarnitine, with a preliminary positive rate of 0.7‰. The second screening was done after 7–14 days for the initial positive neonates, and 171 cases returned to normal, 27 cases were still positive. The positive rate of screening was 0.1‰. Twenty-seven newborns with positive screening results were recalled for genetic tests, of which six rejected to undergo NGS sequencing (Figure 1). The remaining 21 cases were verified by NGS and Sanger sequencing. Ten cases of biallelic variations and one case of single heterozygous mutation were detected of *ACAD8* gene encoding IBD, and seven cases of biallelic variations and one heterozygous mutation of *ACADS* (acyl-CoA dehydrogenase short chain) encoding short-chain acyl-CoA dehydrogenase, one case of biallelic variations of *ACADSB* (acyl-CoA dehydrogenase short/branched chain) gene encoding 2-methylbutyryl-CoA dehydrogenase were detected. No genetic abnormality of *ACAD8* and *ACADS* was detected in the last one case. In conclusion, the prevalence of neonatal IBDD in this area was 1 in 30,299.

### 3.2 Analysis of Biochemical Results

As shown in Table 1, the ten confirmed positive cases all showed characteristic changes of C4 (0.08–0.51), C4/C2 (0–0.05), or C4/C3 (0.04–0.046) by tandem mass spectrometry. In GC-MS, the concentration of 3-hydroxypropionic acid increased in 9 cases and lactic acid increased in 7 cases, all of the cases had the normal concentration of the ethyl malonic acid.

**TABLE 1:** Results of tandem mass spectrometry (MS/MS) and urine GCMS analysis of 10 cases of IBDHD.

No	Sex	Testing time	MS-MS (μmol/L)			GCMS		
			C4 (0.08–0.51)	C4/C2 (0–0.05)	C4/C3 (0.04–0.046)	3-hydroxypropionic acid-2 (0–1.1)	Isobutyrylglycine-1 (0–0.4)	Lactic acid (0.8–4.7)
1	Girl	4 d	1.58 ↑	0.10 ↑	1.35 ↑	—	—	—
		30 d	1.01	0.12 ↑	1.29 ↑	1.75 ↑	0.14	2.82
		2 y	1.73 ↑	0.19 ↑	1.53 ↑	—	—	—
2	Girl	4 d	1.23 ↑	0.09 ↑	0.97 ↑	—	—	—
		18 d	0.89 ↑	0.13 ↑	1.29 ↑	2.56 ↑	0.0	10.27 ↑
		30 d	1.12 ↑	0.13 ↑	1.17 ↑	—	—	—
3	Boy	4 d	1.63 ↑	0.08 ↑	1.48 ↑	—	—	—
		19 d	1.03 ↑	0.14 ↑	2.57 ↑	0.96	1.08 ↑	2.21
4	Girl	5 d	1.87 ↑	0.14 ↑	2.05 ↑	—	—	—
		19 d	1.68 ↑	0.15 ↑	1.54 ↑	6.73 ↑	0.0	93.27 ↑
		3 M	3.33 ↑	0.13 ↑	1.67 ↑	—	—	—
		6 M	3.94 ↑	0.21 ↑	1.57 ↑	—	—	—
5	Girl	3 d	1.53 ↑	0.09 ↑	1.02 ↑	—	—	—
		19 d	1.67 ↑	0.31 ↑	1.88 ↑	8.12 ↑	0.68 ↑	14.96 ↑
		6 M	2.94 ↑	0.05 ↑	1.51 ↑	—	—	—
6	Girl	6 d	1.80 ↑	0.24 ↑	2.28 ↑	—	—	—
		30 d	1.27 ↑	0.23 ↑	1.79 ↑	12.52 ↑	1.0 ↑	5.51 ↑
7	Boy	4 d	1.90 ↑	0.12 ↑	1.64 ↑	—	—	—
		35 d	1.64 ↑	1.14 ↑	1.74 ↑	3.56 ↑	0.4	3.38
		45 d	1.57 ↑	0.08 ↑	1.19 ↑	—	—	—
8	Girl	4 d	1.26 ↑	0.08 ↑	0.84 ↑	—	—	—
		38 d	1.16 ↑	0.14 ↑	1.27 ↑	5.96 ↑	0.0	14.62 ↑
		45 d	1.25 ↑	0.11 ↑	1.13 ↑	—	—	—
		3 y	1.48 ↑	0.20 ↑	1.63 ↑	—	—	—
9	Girl	3 d	1.65 ↑	0.10 ↑	1.49 ↑	—	—	—
		30 d	0.98 ↑	0.15 ↑	1.63 ↑	1.93 ↑	0.0	6.75 ↑
		3 M	1.50 ↑	0.08 ↑	1.03 ↑	—	—	—
		3 Y	1.76 ↑	0.19 ↑	1.57 ↑	—	—	—
10	Girl	3 d	1.46 ↑	0.07 ↑	0.95 ↑	—	—	—
		13 d	0.80 ↑	0.08 ↑	0.78 ↑	6.89 ↑	0.0	14.12 ↑
		45 d	0.91 ↑	0.06 ↑	0.57 ↑	—	—	—
		3 Y	1.20 ↑	0.09 ↑	0.71 ↑	—	—	—

### 3.3 Genetic Testing and Analysis

**FIGURE 2:**
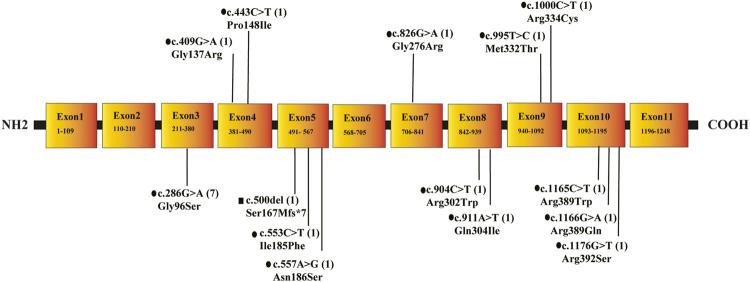
The location, type and frequency of *ACAD8* mutations in 10 patients with IBD. Note: •means missense mutation; ▪indicates a frameshift mutation after.

Sanger sequencing revealed 10 cases of IBDD, all of which were compound heterozygous mutations. The gene of *ACAD8* was found to have 14 mutation sites in six different exons, including 13 missense mutations and one frameshift mutation. Parental verification of seven in the ten IBDD cases confirmed that 7 mutations of them were from their parents (Table 2, Figure 2). Among the mutation sites discovered in this study, six were unreported: c.911A > T, c.904C > T, c.826G > A, c.995T > C, c.1166G > A, c.1165C > T; five (c.286G > A, c.553C > T, c.1000C > T, c.409G > A,c.500del) were judged to be suspected pathogenic after comprehensive evaluation of database query. The clinical significance of the six novel discovered mutations is unknown.

**TABLE 2:** Results of *ACAD8* gene mutation in 10 cases and follow up.

No	Location	Mutation site	Mutation type	Amino acid change	Pathogenicity	Variation type	Verification	Follow-up
P1	Exon8	**c.911A > T**	Compound heterozygous	p.Q304L	VUS	Missense	Maternal	Her brother has the same genotype as her
	Exon8	**c.904C > T**		p.R302W	VUS	Missense	Paternal	
P2	Exon4	c.443C > T	Compound heterozygous	p.P148L	VUS	Missense	—	
	Exon7	**c.826G > A**		p.G276R	VUS	Missense	—	
P3	Exon3	c.286G > A	Compound heterozygous	p.G96S	LP	Missense	—	
	Exon10	c.1176G > T		p.R392S	VUS	Missense	—	
P4	Exon9	**c.995T > C**	Compound heterozygous	p.M332T	VUS	Missense	Paternal	
	Exon10	**c.1166G > A**		p.R389Q	VUS	Missense	Maternal	
P5	Exon3	c.286G > A	Compound heterozygous	p.G96S	LP	Missense	Maternal	
	Exon5	c.553C > T		p.L185F	LP	Missense	Paternal	
P6	Exon3	c.286G > A	Compound heterozygous	p.G96S	LP	Missense	—	Growth retardation, and gradually return to normal
	Exon10	**c.1165C > T**		p.R389W	VUS	Missense	—	
P7	Exon3	c.286G > A	Compound heterozygous	p.G96S	LP	Missense	Maternal	
	Exon9	c.1000C > T		p.R334C	LP	Missense	Paternal	
P8	Exon3	c.286G > A	Compound heterozygous	p.G96S	LP	Missense	Maternal	
	Exon5	c.500del		p.S167Mfs*7	LP	Frameshift	Paternal	
P9	Exon3	c.286G > A	Compound heterozygous	p.G96S	LP	Missense	Paternal	Only mild growth retardation, no other symptoms
	Exon4	c.409G > A		p.G137R	LP	Missense	Maternal	
P10	Exon3	c.286G > A	Compound heterozygous	p.G96S	LP	Missense	Maternal	
	Exon5	c.557A > G		p.N186S	VUS	Missense	Paternal	

Note: Mutation sites not reported in the literature are shown in bold. VUS, means variant of unknown significance; LP, means possible pathogenic variant.

Seven patients who carrying two *ACADS* variants all had increased characteristic changes in tandem mass spectrometry index C4 (0.08–0.51), C4/C2 (0–0.05), and C4/C3 (0.04–0.046). Only five patients participate the GC/MS tests, all the five patients had highly increased EMA (Ethyl malonic acid) and increased 3-hydroxypropionic acid-2 concentration, no patients had increased lactic acid. In addition to the ten confirmed cases, the other patient carried only one heterozygous mutation in *ACAD8* (c.1000C > T).

### 3.4 Follow-Up

Ten IBDD patients were followed up through physical examination to assess their physical and mental development status. It was found that P6 showed mild growth retardation during physical examination at 6 months after birth, and then gradually returned to normal (Table 2); P9 was mild growth retardation when examined at 8 and 10 months after birth. At the age of 3, P9 was 91 cm tall and weighed 11.7 kg. There was still mild growth retardation, but no language or intellectual retardation. No obvious clinical symptoms were seen in the remaining cases (Table 2). In addition, during the follow-up process, it was found that the parents of case P1 gave birth to another son who carries the same two mutations in *ACAD8* as his sister, and also has phenotype of high C4 (Table 2).

Among the six who refused gene testing, C4 acylcarnitine level decreased to normal or close to normal in two cases, and all aspects were normal after follow-up. C4 acylcarnitine was still high in three cases, of which, one case was diagnosed as rickets due to vitamin deficiency, and the rest two were normal. One case was lost during follow-up (Table 3).

**TABLE 3:** Follow-up status of six patients who refused NGS testing.

No	Sex	C4 concentration (μmol/L)		Until 2020
1	Girl	0.78/3 d	0.37/2 m	Normally
2	Girl	1.84/3 d	2.4/1y4 m	Vitamin deficiency, diagnosed rickets, get back to normal
3	Boy	1.34/3 d	0.8/2 m	Loss to follow-up
4	Girl	0.55/3 d	0.51/2 m	Notmally
5	Girl	0.89/3 d	1.12/1 m	Notmally
6	Boy	1.12/3 d	2.65/1 m	Notmally

## 4 Discussion

### 4.1 Prevalence of Isobutyryl-CoA Dehydrogenase Deficiency

IBDD was first reported in 1998 in a two-year-old girl with cardiomyopathy and carnitine deficiency. At 12 months of age, she presented with cardiomyopathy, anemia and carnitine deficiency. Her heart function, growth and development returned to normal after carnitine supplementation. Since then, reports of IBDD cases have gradually appeared abroad, and only a few cases have been reported in Asian countries (Oglesbee et al., 2007; Yoo et al., 2007; Popek et al., 2010; Yun et al., 2015; Santra et al., 2017). Several reports have found that the incidence of IBDD ranges from 1/292,451 to 1/45,466 (Scolamiero et al., 2015). At present, the prevalence of IBDD in China is little known and reported. A survey showed that among 364,545 neonates screened in Quanzhou city, Fujian province, 6 cases (15.4%) were found to have IBDD (Jinping et al., 2019). All these studies show that the incidence of the disease vary greatly from region to region.

In this study, 302,993 newborns were screened by tandem mass spectrometry. Ten IBDD-positive cases were confirmed, and the prevalence of IBDD among newborns in Ningbo city reached 1/30,300. The incidence rates of different regions are also affected by the values set on the detection platform and the formulation of diseases, statistical methods and clinical diagnosis in screening. However, what is certain that as screening and diagnostic techniques improve, rare diseases such as IBDD will be widely known.

### 4.2 Molecular Genetic Etiology of IBDD

One of the characteristics of IBDD is the increase of C4 acylcarnitine. However, in addition to IBDD, short-chain acyl-CoA dehydrogenase (SCAD) deficiency, ethylmalonate encephalopathy, multiple acyl-CoA dehydrogenase deficiency and double iminoglutamic aciduria can also result in increased C4 acylcarnitine by tandem mass spectrometry, and GC-MS biochemical results are not specific. Therefore, tandem mass spectrometry is usually used for IBDD screening, GC-MS is used for auxiliary diagnosis, and a definite diagnosis relies on genetic analysis. So far, *ACAD8* is the only gene reported directly related to IBDD. The *ACAD8* gene is located on chromosome 11q25 and contains 11 exons. So far, more than 22 kinds of mutations of *ACAD8* have been reported, among which missense mutation is the most common type (Yun et al., 2015). In this study, we identified 14 different *ACAD8* variant sites via genetic profiling of IBDD in Ningbo, in which, six have not been reported before. The frequency of c.286G > A variant allele is up to 33.3% (7/21); the frequency of c.1000C > T variant allele is 9.5% (2/21), ranking second. These results suggesting that the two mutations are hot spot or initial mutations of IBDD patients in Ningbo. All the loci mutations detected in this study were missense mutations except one frameshift mutation. Nine unreported mutations were foundafter searching in the disease databases DECIPHER, HGMD, ClinVar and gnomAD. Different from missense mutations in other cases, c.500del mutation of *ACAD8* in case 8 is a deletion mutation. The 167th amino acid of the protein was mutated from serine to methionine (P.S167Mfs*7), leading to premature termination of polypeptide chain synthesis. At present, there is no literature report on this mutation and it is suspected as a pathogenic mutation.

### 4.3 Clinical Research of IBDD

Symptoms of IBDD usually appear in late infancy or childhood, including malnutrition, stunting, dilated cardiomyopathy, epilepsy, and anemia. In 2018, Lin et al. (2018) first introduced the clinical, biochemical and genotypes of 7 IBDD patients in Quanzhou, Fujian province, China. During the follow-up period, five patients were asymptomatic, one adolescent had speech disorder and one newborn had clinical symptoms. Previously, Nygaard et al. (2016b) reported a case of IBDD with obvious clinical symptoms in adulthood, suggesting the risk of asymptomatic children with IBDD developing clinical symptoms in adulthood. Another study of Eleftheriadou et al. (2021) reported a case of an 11-year-old girl diagnosed with IBDD and concluded that IBDD may be associated with autism spectrum disorder. Therefore, further long-term follow-up of the disease is needed to achieve systematic evaluation for the shortest follow-up of the cases was 6 months and the longest was 3 years and 6 months in this study. A long-term follow-up monitoring was recommended for patients diagnosed as hereditary metabolic disease by tandem mass spectrometry screening with short-term clinical symptoms such as IBDD.

In conclusion, the prevalence rate of IBDD among neonates in Ningbo is 1/30,299, among which the c.286G > A site of *ACAD8* has the highest carrying rate, followed by c.1000C > T. The results of this study enriched the mutation spectrum of the *ACAD8* gene; The application of tandem mass spectrometry and NGS sequencing is essential for the diagnosis of IBDD, and long-term follow-up monitoring of IBDD patients is recommended.

## Data Availability

The original contributions presented in the study are included in the article/Supplementary Material, further inquiries can be directed to the corresponding authors.
